# Genetic analyses in mouse fibroblast and melanoma cells demonstrate novel roles for PDGF-AB ligand and PDGF receptor alpha

**DOI:** 10.1038/s41598-020-75774-3

**Published:** 2020-11-09

**Authors:** Julie L. Kadrmas, Mary C. Beckerle, Masaaki Yoshigi

**Affiliations:** 1 Huntsman Cancer Institute, The University of Utah, Salt Lake City, UT 84112 USA; 2 Department of Oncological Sciences, The University of Utah, Salt Lake City, UT 84112 USA; 3 School of Biological Sciences, The University of Utah, Salt Lake City, UT 84112 USA; 4 Department of Pediatrics, The University of Utah, Salt Lake City, UT 84112 USA

**Keywords:** Cytoskeleton, Actin, Cell biology, Cell signalling, Growth factor signalling

## Abstract

Platelet Derived Growth Factor Receptor (PDGFR) signaling is a central mitogenic pathway in development, as well as tissue repair and homeostasis. The rules governing the binding of PDGF ligand to the receptor to produce activation and downstream signaling have been well defined over the last several decades. In cultured cells after a period of serum deprivation, treatment with PDGF leads to the rapid formation of dramatic, actin-rich Circular Dorsal Ruffles (CDRs). Using CDRs as a robust visual readout of early PDGFR signaling, we have identified several contradictory elements in the widely accepted model of PDGF activity. Employing CRISPR/Cas9 gene editing to disrupt the *Pdgfra* gene in two different murine cell lines, we show that in addition to the widely accepted function for PDGFR-beta in CDR formation, PDGFR-alpha is also clearly capable of eliciting CDRs. Moreover, we demonstrate activity for heterodimeric PDGF-AB ligand in the vigorous activation of PDGFR-beta homodimers to produce CDRs. These findings are key to a more complete understanding of PDGF ligand-receptor interactions and their downstream signaling consequences. This knowledge will allow for more rigorous experimental design in future studies of PDGFR signaling and its contributions to development and disease.

## Introduction

Platelet-derived growth factor receptors (PDGFRs) are a critically important family of growth factor-activated receptor tyrosine kinases that broadly direct mitogenic signaling, including both embryonic development and adult tissue repair^1–3^. Aberrant activation of PDGF signaling has also been associated with a variety of different cancers, including melanoma^4^. A striking and robust readout of PDGFR-dependent signaling in cultured cells is the formation of actin-rich Circular Dorsal Ruffles (CDRs)^5–8^. After a period of growth in very low-serum conditions, treatment with PDGF ligand (or other similar growth factors for which the cell has cognate receptors) stimulates the rapid formation of CDRs^9,10^. Within minutes, a phase-dark ring of actin forms at the periphery of the cell. This actin-rich ring rapidly contracts over the dorsal/apical surface (sometimes fracturing into multiple actin rings), internalizing membrane and receptor in a macropinocytotic vesicle. CDRs form dramatically one time per growth factor stimulation and fully resolve on a time scale of approximately 15 min. Additionally, this burst of PDGF signaling allows quiescent cells to initiate the slower downstream responses of both proliferation and remodeling of the actin cytoskeleton so that relatively stationary cells can transition to a migratory state^11^. CDR formation is thought to be a negative feedback mechanism to downregulate/turn off growth factor signaling^12^ and simultaneously deliver cell membrane to the protruding cell edge for growth factor-dependent cell migration^9–11,13^. The formation of CDRs after PDGF ligand stimulation is a facile, sensitive, and reliable method to detect PDGFR activation at early timepoints. Our longstanding interest in the actin cytoskeleton^14–18^ led us to examine these actin-rich CDRs and the PDGFR signaling that elicits them.

The principles governing PDGF-PDGFR binding and signaling in mammalian cells have been well-delineated over the past several decades^1,19,20^. Both the ligands and the receptors are active as dimers. The ligand repertoire consists of four genes encoding four different proteins (PDGF-A, B, C, and D) that dimerize in vivo to bind/activate PDGFRs in stereotypical patterns^21,22^. PDGF-A and PDGF-B ligands are the most widely expressed and characterized, and will be the focus of the studies presented here. Dimerization of these two particular subunits results in functional PDGF-AA, AB or BB ligands. Furthermore, there are two PDGF receptors, PDGFRα and PDGFRβ. In a simplified overview of receptor activation, each receptor subunit has a binding pocket for a PDGF ligand monomer. When a dimeric ligand is bound by two adjacent receptor subunits, the close spatial proximity of their receptor tyrosine kinase domains allows for trans-phosphorylation and full activation of downstream signaling pathways such as PI3 kinase/Akt, Ras/Raf/MEK/ERK, Src and PLC-γ pathways^20^. The receptors signal as PDGFR-αα, -αβ, or -ββ dimers^23,24^.

In a widely accepted model of PDGFR binding and activation, PDGFRα robustly binds both PDGF-A and PDGF-B ligands whereas PDGFRβ is most highly effective at binding PDGF-B^20,24,25^. There is broad consensus that PDGF-AA binds only PDGFR-αα homodimers. PDGF-AB primarily binds PDGFR-αα and -αβ dimers, but may in some contexts also weakly bind PDGFR-ββ^26,27^ at levels speculated to be not physiologically significant^28^. PDGF-BB treatment results in the preferential auto-phosphorylation of PDGFRβ^23^, but PDGF-BB binds all dimeric combinations of the PDGFRs and acts as a ‘universal ligand’^20,29^. As such, PDGF-BB is broadly used to experimentally elicit PDGF signaling as it is effective regardless of which PDGF receptors are expressed.

It has been reported in a wide variety of studies that PDGF-elicited CDR formation and subsequent directed cell migration are activities selectively mediated by PDGFRβ^6–8,20,24,30^. In the course of studying CDR formation, we found that PDGF-AA stimulation (intended to be a negative control, as PDGF-A should not bind PDGFRβ) surprisingly led to substantial and reproducible formation of CDRs in two different cell types. Through additional background reading, we identified another report of PDGF-AA stimulating the formation of CDRs in murine primary lung fibroblasts^31^, suggesting the activity of PDGF-A in eliciting CDRs may be a general phenomenon across a wider variety of cell types. Although the authors did not remark upon this unexpected activity for PDGF-AA, together these provocative results suggest that either PDGF-AA is capable of binding PDGFR-ββ, or that PDGF-AA activated PDGFR-αα homodimers can drive the formation of CDRs. Either of these alternatives is in apparent conflict with established models of PDGFR activity within this field. Because of the critical importance of PDGF signaling in development, cell proliferation and cancer biology, our study seeks to clarify the PDGF ligand-receptor binding profile and the role of PDGFRα signaling in CDR formation.

## Results and discussion

### Mouse embryonic fibroblasts (MEFs) readily form *bona fide* CDRs

**Figure 1:**
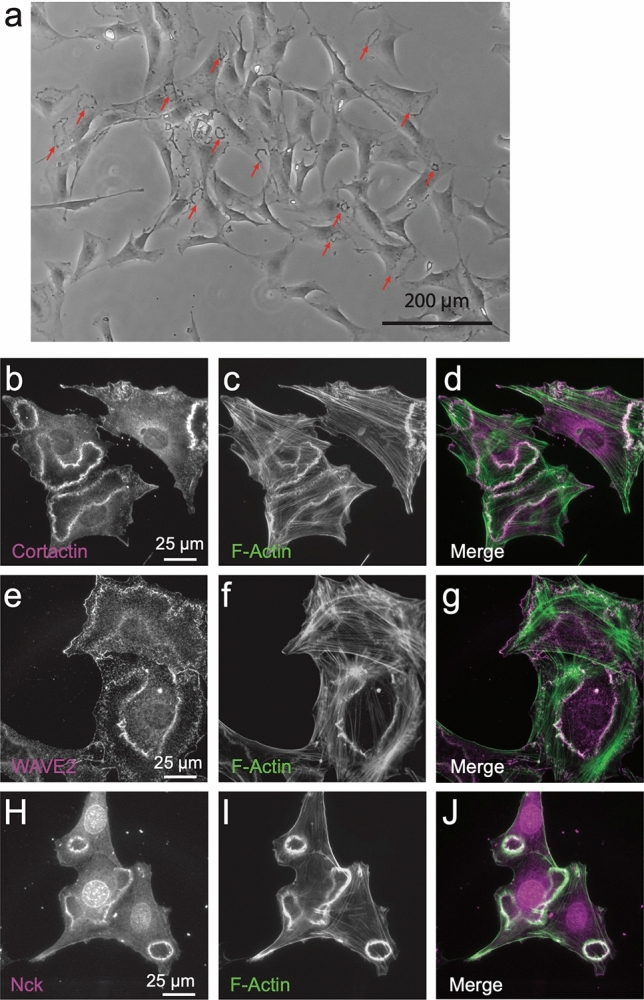
The actin structures elicited with PDGF-BB treatment are *bona fide* CDRs. M28 mouse embryonic fibroblasts were serum-depleted in 0.2% FBS media for 12 h prior to addition of 20 ng/ml PDGF-BB for 6 m. (**a**) A low magnification phase contrase image shows numerous CDRs (select CDRs indicated with red arrows). (**b**–**j**) Immunofluorescence images demonstrating that F-Actin (labeled with phalloidin) (**c**,**f**,**i**) co-localizes with known CDR components cortactin (**b**), WASP2 (**e**) and Nck (**h**) as evidenced in the merge of these signals (overlap appears white) (**d**,**g**,**j**).

After serum deprivation, treatment of MEFs with PDGF-BB leads within minutes to the formation of large actin rings that are visible in a large proportion of the treated cells (Fig. 1a). These F-actin structures show colocalization with established CDR components including cortactin^32^ (Fig. 1b–d) and WAVE2^33^ (Fig. 1e–g) which regulate actin dynamics, as well as the signaling adaptor Nck^7^ (Fig. 1h–j). These data support that the actin structures we observe with the universal ligand PDGF-BB are indeed CDRs. We next wanted to determine the PDGFR expression profile for the cell lines used in this study, and quantify the response to a wider variety of PDGF ligands.

### Clonal fibroblast and melanoma cell lines express both PDGFRα and β, and exhibit receptor activation and CDR formation after treatment with PDGF-AA, AB and BB ligands

**Figure 2:**
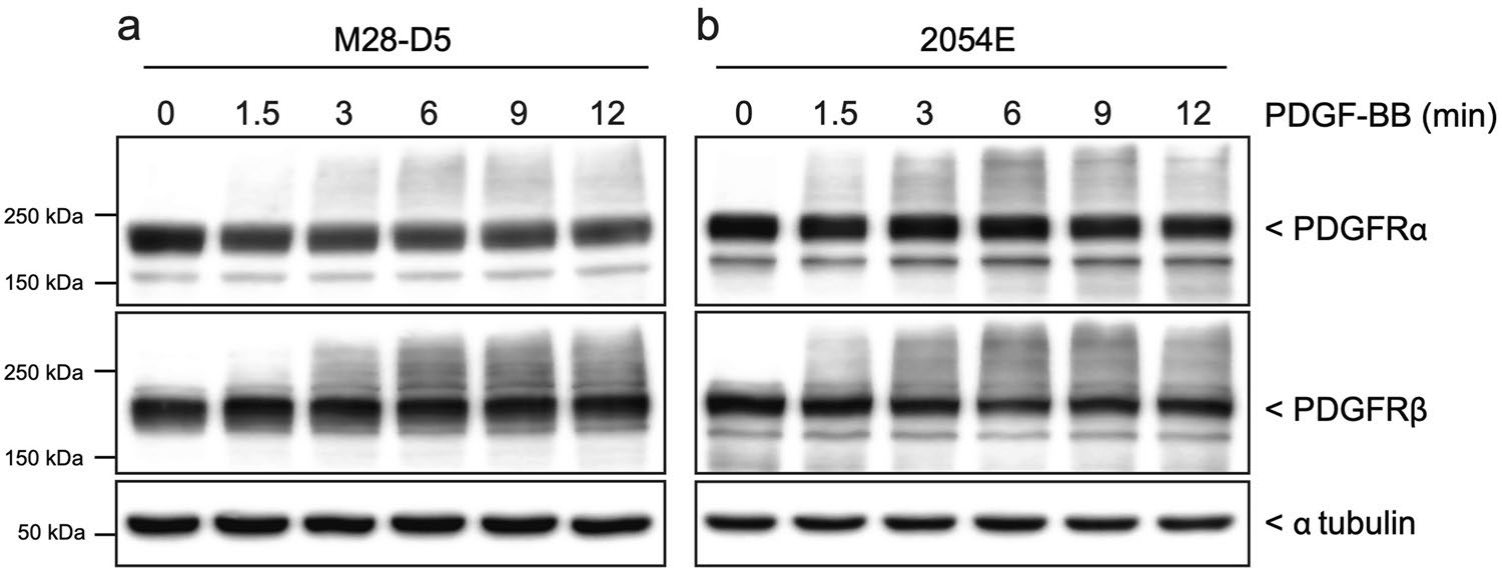
Treatment with PDGF ligands activates both PDGFRs expressed in M28-D5 fibroblasts and 2054E melanoma. Cropped immunoblots show that M28-D5 fibroblasts (**a**) and 2054E melanoma cells (**b**) express both PDGFRαα and PDGFRβ. In a time course after treatment with 20 ng/ml PDGF-BB, both receptor isotypes exhibit an increase in high molecular weight signal. Tubulin is used as a loading control. Full-length images of cropped immunoblots are presented in Supplementary Figure S1.

**Figure 3:**
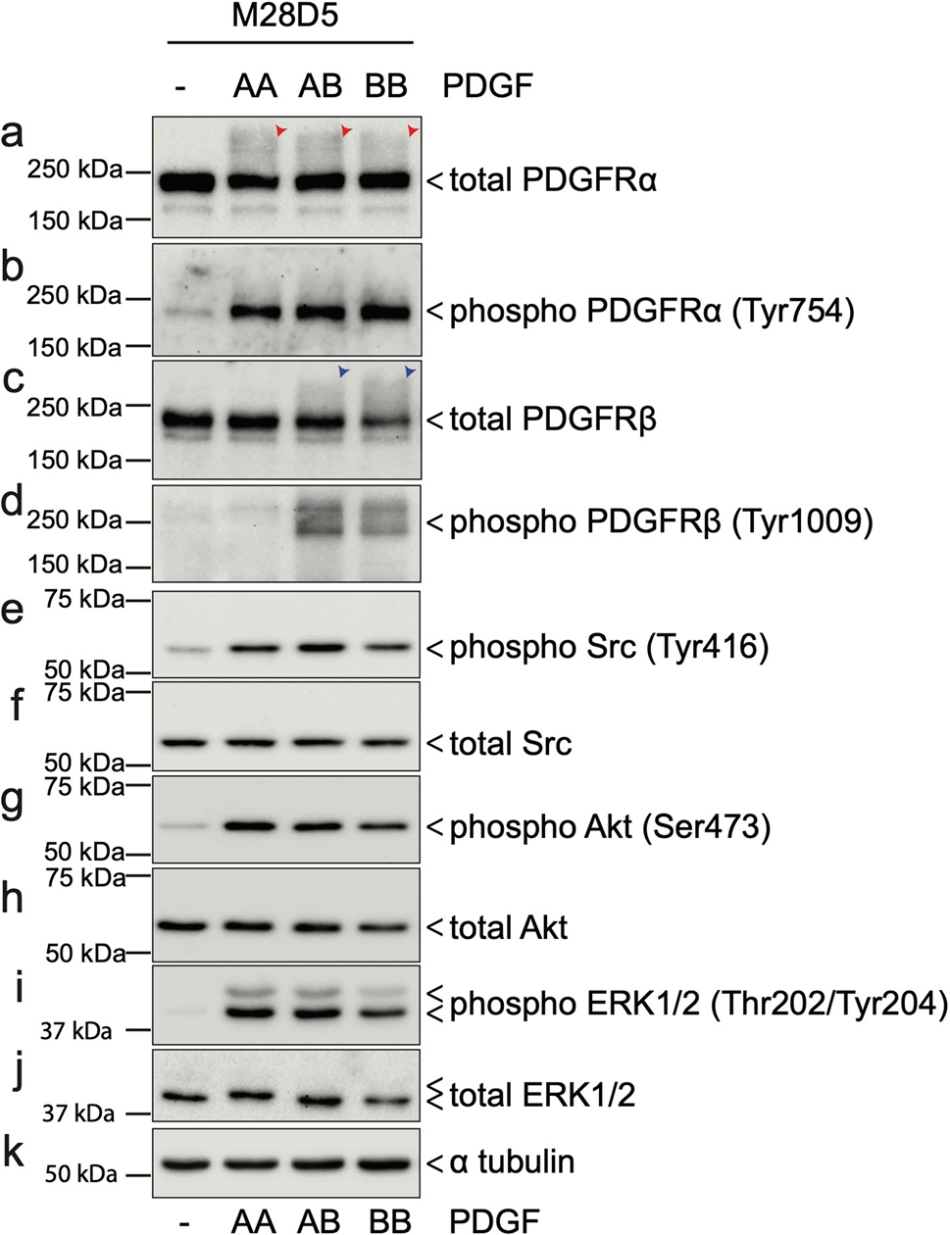
Diffuse high-molecular weight signals in PDGFRα and PDGFRβ immunoblots correlate with receptor phosphorylation and downstream pathway activation. M28-D5 fibroblasts were stimulated by 20 ng/ml of PDGF-AA, AB, or BB ligands for 6 m after serum depletion in 0.2% FBS media for 12 h. Immunoblots show (**a**) robust PDGFRα phosphorylation in the total PDGFRα immunoblot (red arrowheads) in the high-molecular weight (> 250 kDa) regions which is well correlated with (**b**) the robust increase in Tyr754 phosphorylation of PDGFRα. Likewise, the high-molecular weight signals in (**c**) total PDGFRβ immunoblot (blue arrowheads) are well correlated with (**d**) the Tyr1009 phosphorylation of PDGFRβ. (**e**–**j**) Phosphorylation of several key signaling molecules downstream of PDGFR was confirmed by phospho-specific antibodies to detect canonical phosphorylation/activation sites. In response to all AA, AB, and BB PDGF ligands, Src, Akt, and ERK were robustly phosphorylated while total levels of these proteins remained constant. (**k**) Alpha tubulin was used as a loading control.

The cell lines used in the remainder of these studies are derived from M28 MEFs and 2054 mouse melanoma cells. The M28 MEFs were previously isolated in our lab from a wild type C57BL/6 mouse^14^. The 2054 melanoma cells were transformed via an NRAS oncogene in a mouse model of melanoma^34^. They represent both ‘normal’ and cancer cells that express both of the PDGFRs. Both of these starting cell lines exhibit somewhat heterogeneous cell morphology in culture. Therefore, we isolated single cell clones from each cell line by limiting dilution in 96-well plates. Multiple fibroblast and melanoma clonal cell lines were established. The clonal cell lines selected for further study, M28-D5 and 2054E, were chosen because they robustly express both PDGFRα and PDGFRβ as evidenced by immunoblots (Fig. 2). Furthermore, after 12 h of growth in media containing very low amounts of fetal bovine serum (0.2%), treatment with the universal ligand PDGF-BB triggers an increase in diffuse high-molecular weight signal observed in both PDGFRα and PDGFRβ immunoblots at time points ranging from 90 s to 12 m (Fig. 2). This high molecular weight smear is unlikely to represent receptor dimers, as the immunoblots were conducted under reducing conditions. Moreover, it is unlikely to represent simple receptor phosphorylation, as the small size of phosphate groups should be unable to affect such a large shift in molecular weight. However, as demonstrated in Fig. 3, the increase in signal in the high-molecular weight regions of PDGFRα and PDGFRβ immunoblots correlates well with increased phosphorylation of these receptors detected by site specific phospho-antibodies (PDGFRα (Tyr754) and PDGFRβ (Tyr1009)), regardless of which PDGF ligand is employed (Fig. 3 a-d). Notably, the PDGFRβ shift is largely absent after treatment with PDGF-AA, which does not appreciably bind/activate PDGFRβ (Fig. 3c,d). Moreover, the increase in high molecular weight signal also correlates with the phosphorylation/activation of downstream signaling proteins such as Src (Fig. 3e,f), Akt (Fig. 3g,h) and ERK1/2 (Fig. 3i,j). Thus, we demonstrate that an increase in high molecular weight signal in PDGFR immunoblots can be used as an all-purpose surrogate for receptor activation.

**Figure 4:**
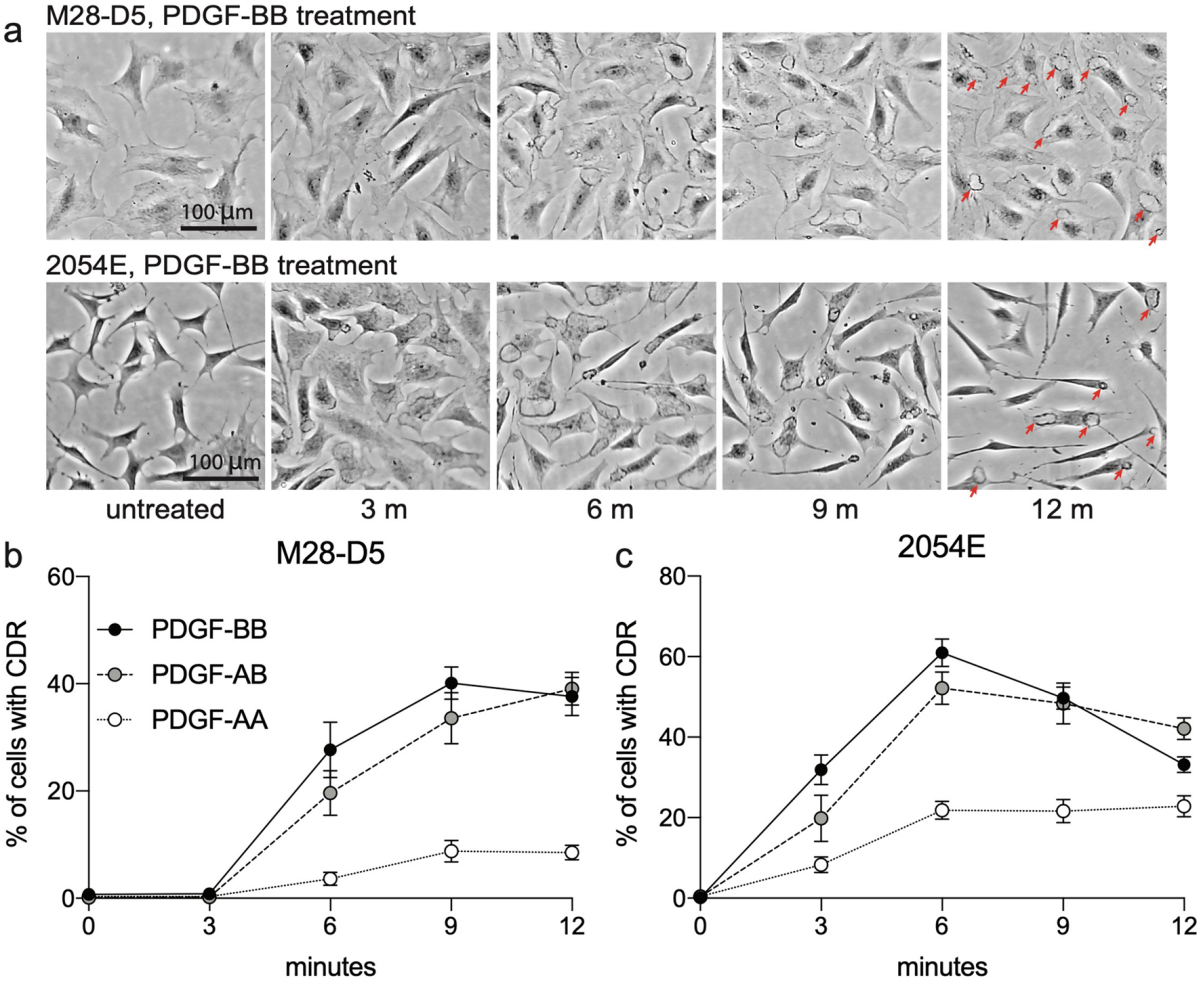
Treatment with PDGF ligands stimulates CDR formation downstream of PDGFR activation. (**a**) Representative phase contrast images at various time points after PDGF-BB addition. Red arrows in the 12 m images highlight the presence of CDRs. (**b**,**c**) Quantification of the frequency of CDRs observed in M28-D5 fibroblasts (**b**) and 2054E melanoma cells (**c**) at various times after the addition of PDGF-AA (open circles), PDGF-AB (grey circles) or PDGF-BB (black circles).

After the receptor activation evident in immunoblots (Fig. 2), robust formation of CDRs is observed in both cell lines (Fig. 4). In a time-course of phase contrast images after treatment with PDGF-BB, the actin ring of the CDR is readily evident after 6 min in M28-D5 and after 3 min in 2054E (Fig. 4a). Quantification of the frequency of CDRs after treatment with various PDGF ligands is shown in Fig. 4b,c. The universal ligand PDGF-BB elicits CDRs in approximately 40% of M28-D5 fibroblasts and 60% of 2054E melanoma cells. PDGF-AB treatment induces CDR formation in both cell lines at levels similar to PDGF-BB. PDGF-AA also reproducibly triggers considerable CDR formation in both cell types, but at a reduced frequency.

### Genetic elimination of Pdgfra has minimal effect on Pdgfrb expression in M28-D5 and 2054E cells

**Figure 5:**
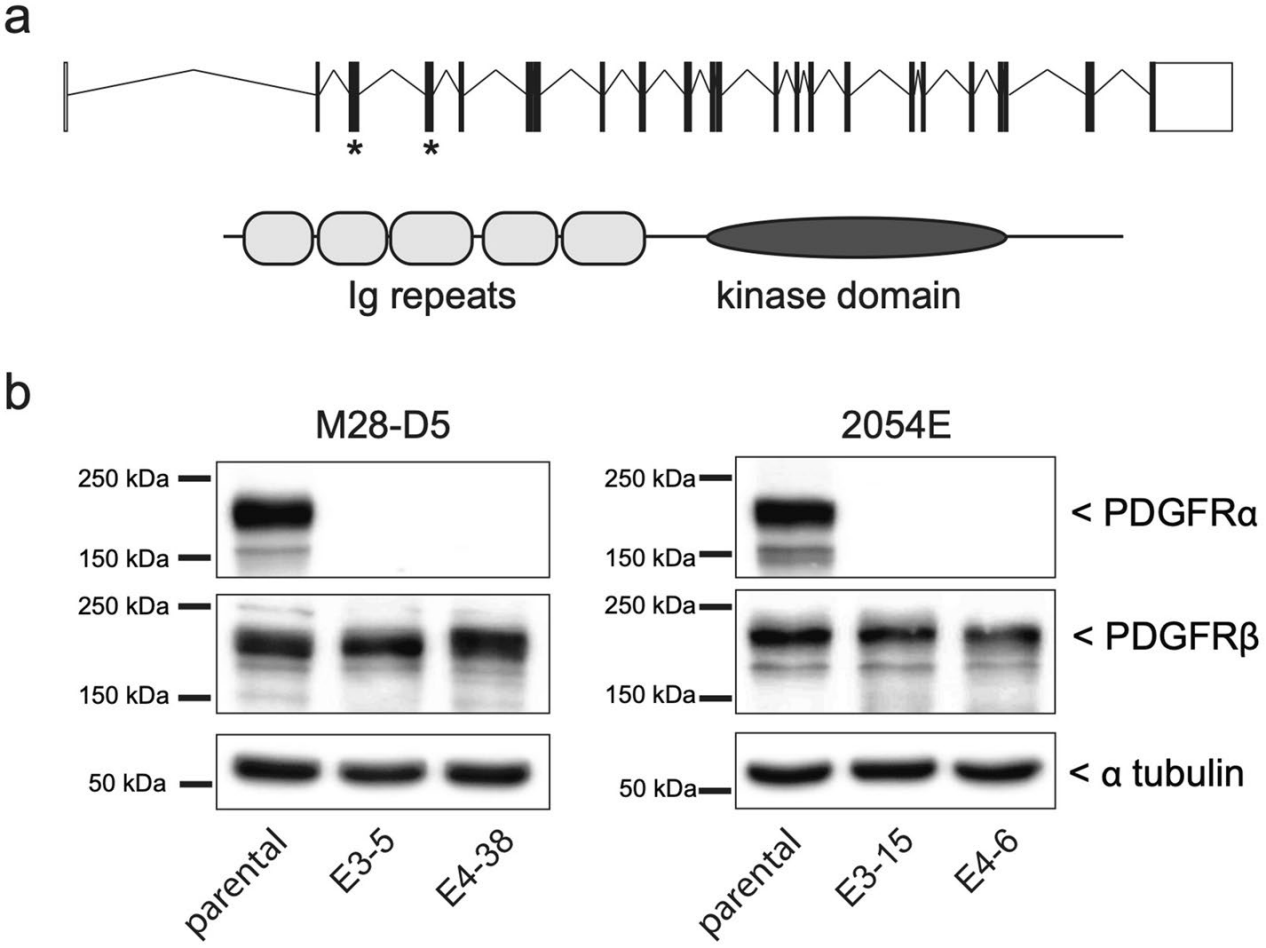
CRISPR/Cas9 gene editing disrupts PDGFRα expression. (**a**) Top: Schematic diagram of the mouse *Pdgra* gene, with exons depicted as boxes and introns as connecting lines. Asterisks indicate the exons targeted for CRISPR/Cas9 cleavage. Bottom: Domain structure of the mouse PDGFRα protein. (**b**) Cropped immunoblots showing the PDGFR expression profile of both the parental M28-D5 and 2054E cell lines, as well as the *Pdgfra*^*−/−*^ cell lines derived from them. Cells were grown under standard culture conditions. Alpha-tubulin is used as a loading control. Full-length images of the cropped immunoblots are presented in Supplementary Figure S1.

To clarify both the PDGF ligand-receptor binding profile and rigorously test the role of PDGFRα signaling in CDR formation, we genetically disrupted *Pdgfra* in M28-D5 fibroblasts and 2054E melanoma cells using CRISPR-Cas9 gene editing. The *Pdgfra* gene contains 23 exons that encode a 1089 amino acid PDGFRα protein (Fig. 5a). Mutations engineered into either exon 3 or 4 produce disruptions early in the *Pdgfra* coding sequence (in the extracellular immunoglobulin repeats) and are predicted to fully disrupt expression of functional protein. Small guide RNAs directed to either exon 3 or exon 4 of the mouse *Pdgfra* gene were transiently expressed along with Cas9 nuclease to target double stranded DNA breaks with imprecise repair. Clones were screened for the absence of PDGFRα expression via immunoblots (Fig. 5b) with a polyclonal antibody raised against PDGFRα Leu25-Glu524. The genomic DNA of clones that lacked detectable PDGFRα protein expression was further analyzed to ensure that no wild type *Pdgfra* DNA sequence was present. Clones with small 1–2 bp insertions or deletions on both chromosomes were selected for further analyses (Table 1). In these selected cell lines, the absence of PDGFRα had minimal impact on the expression of PDGFRβ (Fig. 5b).

**Table 1:** Genetic lesions present in the *Pdgfra* null cell lines analyzed.

Pdgfra null cell line	Exon targeted	Genetic lesion at PAM site
M28–D5^E3–5^	3	2 bp deletion (GT)
		2 bp deletion (GT)
M28–D5^E4–38^	4	1 bp insertion (T)
		2 bp deletion (GT)
2054E^E3–15^	3	1 bp deletion (G)
		2 bp deletion (GT)
2054E^E4–6^	4	1 bp insertion (T)
		2 bp deletion (GT)

In the experiments that follow, the *Pdgfra*^*−/−*^ cells were directly compared to the parental cell lines from which they were derived. Because the *Pdgfra* gene was disrupted using two independent small guide RNAs, phenotypes that are observed with both targeting strategies are considered to be *bona fide*, and are unlikely due to off-target effects. Rescue experiments with plasmid-expressed PDGFRα are therefore not necessary. The *Pdgfra*^*−*/*−*^ cells to be analyzed are M28-D5^E3–5^ and 2054E^E3–15^ (disrupted in exon 3), M28-D5^E4–38^ and 2054E^E4–6^ (disrupted in exon 4).

### PDGF-AA binds and activates PDGFRα homodimers and stimulates CDR formation

**Figure 6:**
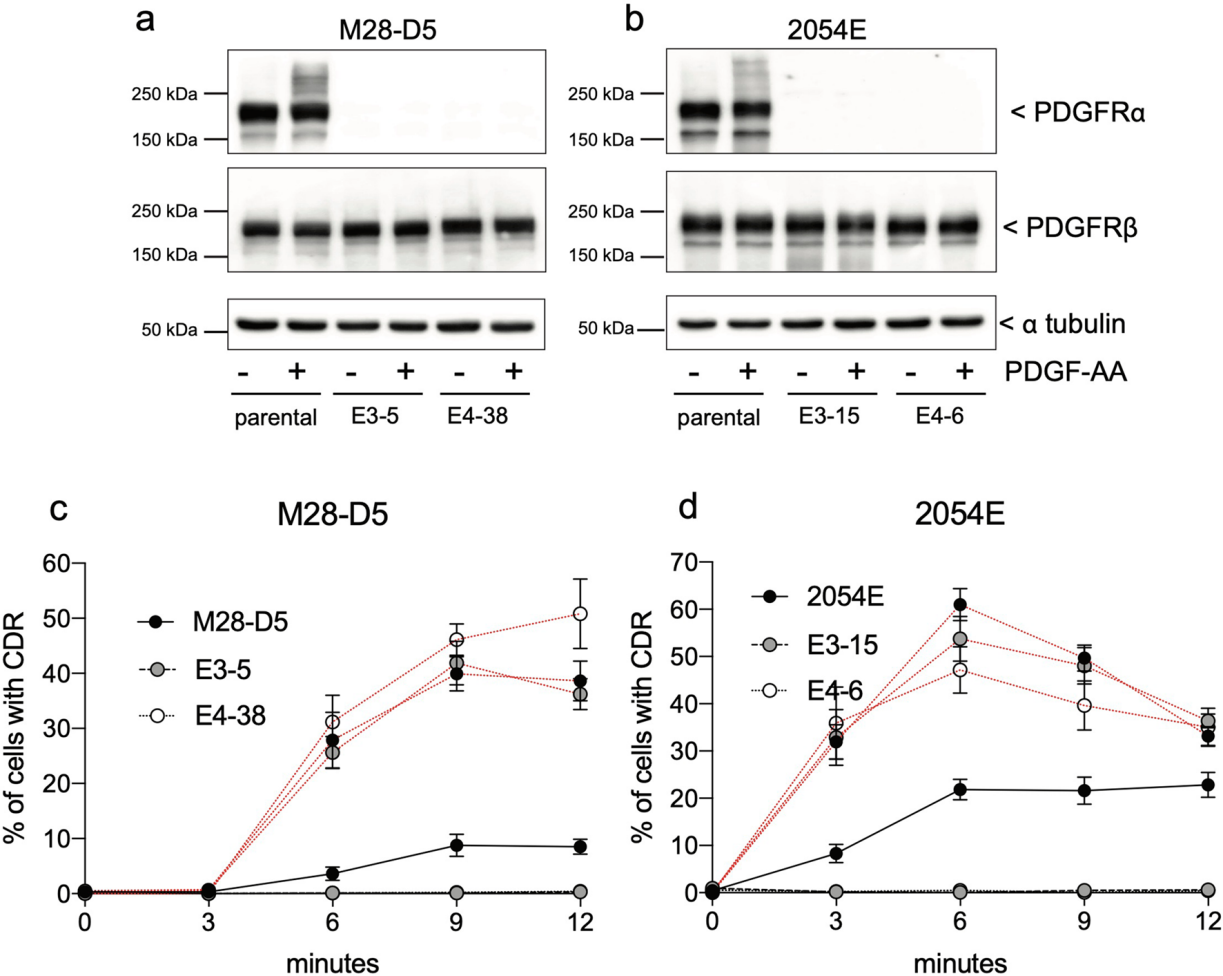
PDGFRα is required for a response to PDGF-AA treatment. (**a**,**b**) Cropped immunoblots demonstrate a loss of PDGFRα expression in the *Pdgfra* null cells that were constructed from M28-D5 (**a**) and 2054E (**b**) with no gross compensatory changes in PDGFRβ expression. Treatment of these cells with 20 ng/ml PDGF-AA for 9 m leads to the activation of PDGFRα in the parental cell lines (increase in high molecular weight signal) but has little effect on PDGFRβ activation. Full-length images of cropped immunoblots are presented in Supplementary Figure S1. (**c**,**d**) Quantification of the frequency of CDR formation at various time points after PDGF-AA (black lines) compared to PDGF-BB (red lines) treatment in both parental and *Pdgfra* null fibroblasts (**c**) and melanoma cells (**d**) targeted in both exon 3 and exon 4.

To more fully understand the activity of the PDGF-AA ligand, we compared PDGF-AA stimulation of the parental cell lines with matched *Pdgfra* deficient cells. Treatment with PDGF-AA preferentially activates PDGFRα, as seen by the high molecular weight shift in the parental cell lines in PDGFRα immunoblots (Fig. 6a,b). No significant shift/activation was evident in PDGFRβ immunoblots in any of the cell lines treated with PDGF-AA (Fig. 6a,b). Concurrently, PDGF-AA elicited CDR formation in approximately 10% of parental M28-D5 fibroblasts (Fig. 6c, black lines) and 25% of 2054E melanoma cells (Fig. 6d, black lines). However, treatment with PDGF-AA does not drive the formation of CDRs in any of the matched *Pdgfra*^*−/−*^ cell lines, regardless of whether they are fibroblasts or melanoma, or the exon targeted for gene disruption (Fig. 6c,d, black lines). The complete absence of CDRs in PDGF-AA treated *Pdgfra*^*−/−*^ cells together with undetectable PDGFRβ activation by immunoblot suggests that PDGF-AA activates PDGFRα homodimers to produce CDRs. As shown for comparison (Fig. 6c,d, red lines), CDR formation in the parental cells with universal ligand PDGF-BB is more efficient. However, PDGF-BB dependent CDR formation is largely unaffected by the genetic elimination of *Pdgfra* because it can continue to activate PDGFRβ. Our data support that efficient PDGF-AA dependent CDR formation requires the presence and activity of PDGFRα, arguing against the idea that PDGFRβ is solely responsible for CDR formation. However, the possibility remains that PDGF-AA may bind and activate PDGFRβ at some low level to produce a portion of the observed CDRs. In future work, PDGF-AA dependent CDR formation in cells where *Pdgfrb* is genetically eliminated could provide additional evidence to test this idea.

### PDGF-AB elicits physiologically relevant signaling and CDR formation via activation of PDGFR-ββ

**Figure 7:**
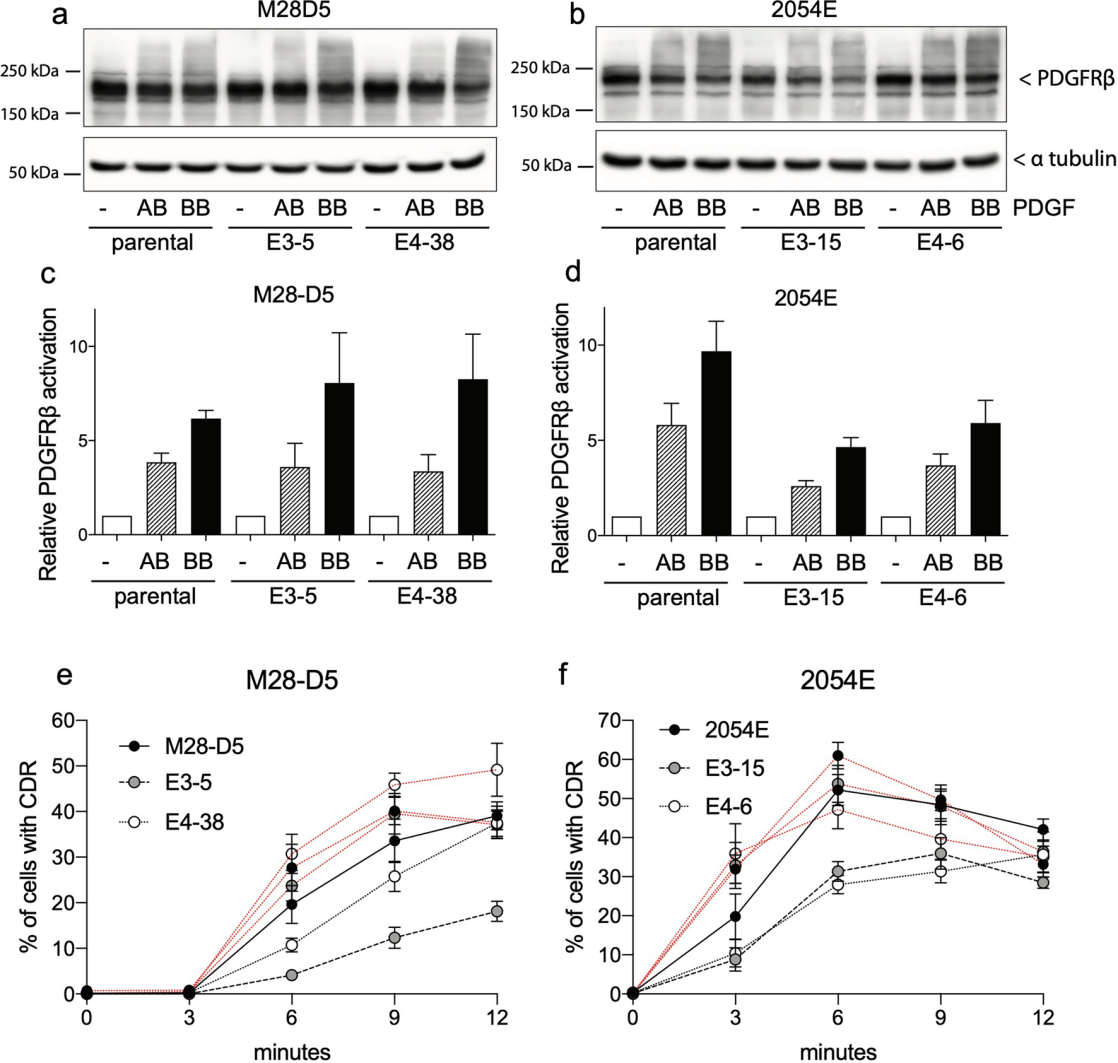
PDGF-AB elicits physiologically relevant signaling via activation of PDGFR-ββ. (**a**,**b**) Cropped PDGFRβ immunoblots for both parental and PDGFRα null fibroblasts (**a**) and melanoma cells (**b**). Cells were either untreated or treated for 9 m with 20 ng/ml PDGF-AB or PDGF-BB. Receptor activation is evident as an increase in high molecular weight signal. Cropped alpha-tubulin immunoblots demonstrate equal loading. Full-length images of cropped immunoblots are presented in Supplementary Figure S1. (**c**,**d**) Quantification of PDGFRβ activation observed in the immunoblots, relative to the untreated control for both fibroblasts (**c**) and melanoma cells (**d**). The bar graph represents the mean for the quantification of 5 independent experiments. Error bars reflect SEM. (ImageJ Ver 1.50b imagej.nih.gov) (**e**,**f**) Time course showing the frequency of CDR formation after treatment with PDGF-AB (black lines) or PDGF-BB controls (red lines) in fibroblasts (**e**) and melanoma cells (**f**).

It is widely accepted that PDGF-AB can robustly activate PDGFR-αα and -αβ, but it is inefficient at activating PDGFR-ββ homodimers^26–28^. We directly test this claim by comparing PDGF-AB versus PDGF-BB dependent signal activation in the M28-D5 and 2054E parental cells (which contain all combinations of PDGF receptors) to that in the matched *Pdgfra*^*−/−*^ cells (which can form only PDGFRβ homodimers). In the M28-D5 and 2054E parental cell lines, treatment with either PDGF-AB or PDGF-BB clearly stimulates an increase in activated, high molecular weight PDGFRβ (Fig. 7a,b). Quantification of this activation (Fig. 7c,d) shows that PDGF-BB is most efficient at receptor activation, but PDGF-AB is able to stimulate approximately half the level of PDGFRβ activation in all the cell lines examined. Furthermore, PDGF-AB stimulates the formation of CDRs at a frequency comparable to PDGF-BB treatment in both of the parental cell lines (Fig. 7e,f black lines vs red lines). In matched cells lacking PDGFRα, CDR formation remains relatively unaffected after PDGFR-BB treatment (Fig. 7e,f). In the M28-D5 fibroblasts, targeting *Pdgfra* in exon 3 results in a twofold reduction in PDGFR-AB dependent CDR formation, yet a sizable fraction of cells do continue to exhibit CDRs (Fig. 7e). Targeting *Pdgfra* in exon 4 had no substantial impact on the ability of these fibroblasts to respond to PDGF-AB (Fig. 7e). In 2054E melanoma cells, loss of *Pdgfra* resulted in 15–35% reductions in the frequency of PDGF-AB dependent CDR formation at 9–12 min, regardless of the exon targeted (Fig. 7f). Notably, the reductions observed in PDGF-AB dependent PDGFRβ activation and CDR frequency are at most twofold. This is in contrast to the accepted model that only very high concentrations of PDGF-AB can activate PDGFR-ββ^28^. At 20 ng/ml PDGF-AA, we observe much higher receptor activation and CDR formation than would be expected based on prior studies. Thus, we have demonstrated conditions under which PDGF-AB is clearly capable of activating PDGFRβ homodimers, refuting the idea that PDGF-AB cannot efficiently elicit signaling via PDGFR-ββ.

### Revised model for PDGF-PDGFR binding and signaling

**Figure 8:**
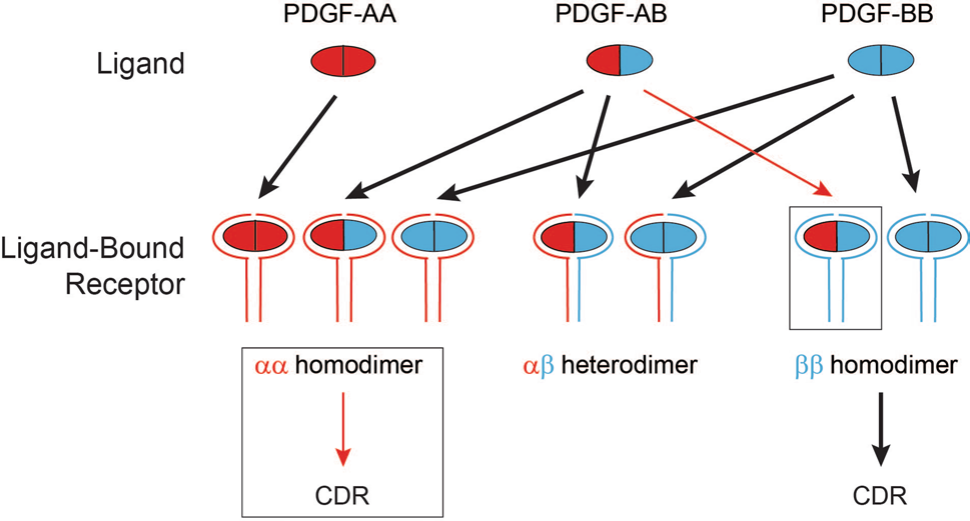
Revised model for PDGF-PDGFR binding and signaling. Canonical ligand-receptor interactions and those responsible for eliciting CDR formation are indicated with black arrows. Novel insights are boxed and indicated with red arrows. The relative strength of the activity is approximated by the weight of the arrow.

Collectively these new data have allowed for revision of PDGF receptor-ligand binding interactions. Previously established interactions and CDR activity are shown in Fig. 8 with black arrows. Red arrows and boxed portions indicate revisions to this scheme based on our studies. Notable findings include: (1) PDGFRα can elicit CDRs, in contradiction to the assertion that PDGFRβ is solely responsible. (2) PDGF-AB can robustly activate PDGFRβ homodimers, and thus has a broader spectrum of efficient receptor binding than previously appreciated. What allowed us to identify these discrepancies? Early studies of PDGF signaling were rigorously conducted, but subject to the limitations of the techniques available at that time. To study the activities of PDGFRα and PDGFRβ in isolation, several different strategies were employed. (1) The receptors were exogenously expressed in cells that did not normally express them, such as Porcine Aortic Endothelial cells^6^. Such studies rely on all relevant downstream signaling molecules being present, and are most compelling when ligand-dependent PDGFR activity is observed. However, when PDGF ligands do not activate downstream signaling via the exogenously expressed PDGFR, this might be due to non-native composition of the experimental system. (2) Reduction of PDGFR protein in cells that normally express them was carried out with RNAi or by depletion of cell surface PDGFR through CDR internalization^24^. Frequently in such studies, levels of remaining total or surface PDGFR were either unmeasured or measured with qualitative methods (northern or western blots). An obvious limitation of depletion is that residual PDGFR retains the capacity to signal. In this scenario, reduced levels of signaling can be challenging to unambiguously interpret. (3) Alternatively, cell lines empirically found to lack PDGFR expression were compared to similar cell lines that express the PDGFR. One example of this is 3T3 fibroblasts derived from the Patch mutant mouse in which PDGFRα (but not PDGFRβ) expression is missing. Treatment of these cells with PDGF-AB resulted in dramatic reductions in signaling compared to 3T3 fibroblasts derived from wild type mice^28^. In this context, it is unclear whether PDGF-AB poorly activates PDGFRβ homodimers, or if the low activity is a result of additional differences in the Patch genetic background. Germ line PDGFRα and β knock-out mice constructed in past studies are embryonic lethal^35–39^. Unfortunately, embryonic cell lines derived from full knock-out mice have the same difficulties in matching the control cell lines derived from physically different animals. Each of the experimental systems previously used to study individual PDGFR activity had limitations. Full genetic knock-out of individual PDGFRs via CRISPR-Cas9 editing in clonal cell lines and comparison to their fully-matched control cell lines have allowed for more definitive studies, identifying CDR formation downstream of PDGFRα, and robust interaction between PDGF-AB and PDGFRβ homodimers.

## Materials and methods

### Reagents, cell lines and culturing conditions

Reagents include Rat recombinant PDGF-AA, PDGF-AB, and PDGF-BB (R&D Systems). Cell lines include wild type M28 MEFs originally isolated from a C57BL/6 mouse in our lab^14^. The study protocol was approved by the University of Utah Institutional Animal Care and Use Committee. All experiments performed were in accordance with relevant guidelines and regulations. Additional cell lines are M28-D5, a single cell clone derived from M28, and the *Pdgfra*^*−/−*^ MEFs derived from M28-D5 as described; and 2054E, a single cell clone derived from 2054 melanoma cells^34^ (provided by Sheri Holmen and Matt VanBrocklin), plus the *Pdgfra*^*−/−*^ melanoma lines derived from 2054E, also as described. Unless otherwise indicated, cells were grown in high glucose Dulbecco's Modified Eagle Medium (Gibco) supplemented with Penicillin–Streptomycin (Thermo Fisher Scientific) and 10% fetal bovine serum (FBS) under standard culture conditions (37 °C, 5% CO_2_).

### Immunofluorescent cell imaging

Cells were plated on uncoated coverslips, and grown as indicated. Prior to imaging, cells were fixed 15 min in 3.7% paraformaldehyde, and permeabilized in 0.2% Triton X-100 for 5 min. Cells were incubated with either Phalloidin (1:200, to detect F-Actin) or primary antibodies to Cortactin (1:400, Upstate Biotech), WAVE2 (1:400, Cell Signaling) or Nck (1:400, BD Biosciences). AlexaFluor conjugated secondary antibodies (1:8000 to 1:10,000, Thermo Fisher Scientific) were then used to visualize protein localization. Cell images were captured using a Zeiss Axioplan 2 with 40X, NA 0.75 objective and Zeiss AxioCam CCD Camera. Pseudocolor images were created in Photoshop using green-magenta color scheme in which colocalization appears white.

### Immunoblotting

Cells were rapidly washed in ice-cold PBS twice, and lysed in modified RIPA buffer containing 1% NP-40, 0.2% SDS, EGTA (1 mM), Tris pH 8.0 (50 mM), NaCl (20 mM), beta-glycerophosphate (10 mM), NaF (20 mM). All cell lysis procedures were performed on ice. Sample viscosity due to DNA extraction was cleared by 26G syringe. Cell debris was removed by centrifugation at 16,000×g for 5 min at 4 °C. Protein concentration was measured using DC Assay (Bio-Rad). Samples were denatured and reduced in Laemmli sample buffer with 50 mM DTT followed by incubation in 95 °C for 4 min. Proteins were resolved in fixed concentration SDS-PAGE system (8% for PDGFR blots, 10% for other blots), and electro-blotted to PVDF membrane in Tris Glycine/Methanol buffer system. Protein transfer was monitored using copper phthalocyanine tetrasulfonic (CPTS) acid stain. Blot membranes were blocked in 4% non-fat milk dissolved in TBS-Tween wash buffer for 1 h. Primary antibodies used for western immunoblots include: goat anti-mouse PDGFRα and PDGFRβ (each at 1:2500, R&D Systems), rabbit anti-phospho-PDGFRα (Y754, 1:2000), phospho-PDGFRβ (Y1009, 1:2000), phospho-Src (1:2000), total Src (1:2000), phospho-ERK1/2 (1:2000), total ERK1/2 (1:2000), phospho-Akt (1:2000), total Akt (1:2000, Cell Signaling Technology), and mouse anti-alpha tubulin (1:4000, Invitrogen). HRP conjugated secondary antibodies were employed (1:6000 to 1:10,000,GE Healthcare and/or Jackson ImmunoResearch Laboratories). Signals were detected using SuperSignal West Femto (Thermo Fisher Scientific) and captured by KwikQuant Imager hardware (Kindle Biosciences). Densitometry was performed using ImageJ (Ver 1.50b, NIH, ImageJ.nih.gov). All immunoblot images presented are representative of the results observed from at least 3 independently conducted experiments.

### Quantification of CDR formation

Experiments were conducted in 12-well plates, with separate wells for each treatment (PDGF-AA, AB, and BB) and timepoint (0, 3, 6, 9, 12 min). Where indicated, cells were cultured in 0.2% FBS for 12 h, then 20 ng/mL of the indicated PDGF ligand was applied. This is typical of the concentration of PDGF routinely used to induce CDR formation in a variety of different cell types^5–7,13,40–45^. Cells were rapidly fixed with 3.7% paraformaldehyde at the designated timepoints after PDGF ligand addition. To quantify CDR formation, phase contrast images were acquired for three fields of view in each well. In each image, total cell number was counted and compared to the number of cells exhibiting CDRs. Each experiment was conducted 3 independent times. The fraction of cells exhibiting CDRs was plotted as the mean percentage ± SEM from nine images for each data point. The total number of cells counted for each data point ranged from approximately 300 to 1000 cells, with a mean of 641 cells counted.

### Generating Pdgfra^*−*/*−*^ cells via CRISPR/Cas9 genome editing

In M28-D5 MEFs and 2054E melanoma cells, we disrupted the *Pdgfra* gene via CRISPR/Cas9 genome editing using plasmids that transiently express small guide RNAs that independently target exon 3 (GGGTCGTCTTCTTCAGACAT) or exon 4 (GGTCATCCCGAGAGGCACAA) of the mouse *Pdgfra* gene. Single cell clones were transiently selected for the presence of the CRISPR plasmid (Puromycin selection) and screened for lack of detectable PDGFRα protein expression by immunoblot. Lines showing absent PDGFRα protein expression were further analyzed. Genomic DNA was prepared and a portion of the *Pdgfra* gene surrounding the cleavage site was sequenced to confirm disruption on both chromosomes, indicating no wild type PDGFRα gene product can be expressed. PCR primers for amplification of the targeted region were: Exon 3: forward 5′ attcaatggctgtccctttc 3′; reverse 5′ ggtctaggagggccctgcaa 3′; Exon 4: forward 5′ cttctctctctctttaaaat 3′; reverse 5′ ctctcacttagagaggtgaa 3′. Low passage *Pdgfra*^*−/−*^ clonal cell lines were directly compared to the matched parental cell line. When both the Exon 3 and Exon 4 target sites produce similar phenotypes in the *Pdgfra*^*−*/*−*^ cells, we attribute phenotypes to the loss of *Pdgfra*, rather than off-target effects.

### Quantification of PDGFRβ activation

In PDGFRβ immunoblots of untreated cells or cells treated with PDGF-AB or PDGF-BB, signal intensification in the area above the main 190 kDa PDGFRβ band was quantified using ImageJ (ver 1.50b, NIH, ImageJ.nih.gov). A region of interest was designated and compared both to an adjacent control area, and to the untreated control sample. Signal intensity is plotted as a fold-change relative to the untreated cells and reflects the mean ± SEM from 5 independent experiments.

## Supplementary information

Supplementary Information.

## Data Availability

All data generated or analyzed during this study are included in this published article and its Supplementary Information files.
