# Supplementary material for: Genetic analyses in mouse fibroblast and melanoma cells demonstrate novel roles for PDGF-AB ligand and PDGF receptor alpha
Source: Sci Rep. 2020 Nov 9;10:19303. doi: 10.1038/s41598-020-75774-3 (PMC7653911; doi:10.1038/s41598-020-75774-3)
Supplement: Supplementary file 1 — Supplementary Information. [file 41598_2020_75774_MOESM1_ESM.pdf]

Supplementary Figure S1, Full-length images of cropped immunoblots in figures

Figure 2a, M28-D5, PDGFRα

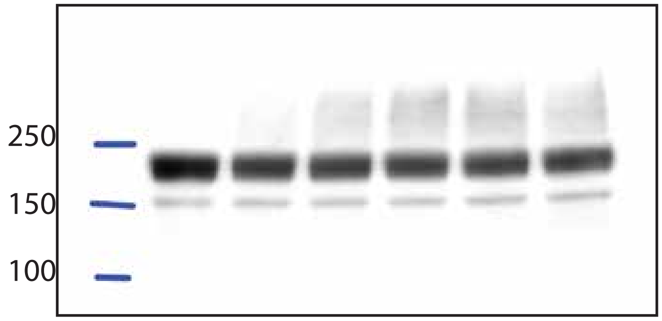

Figure 2b, 2054E, PDGFRα

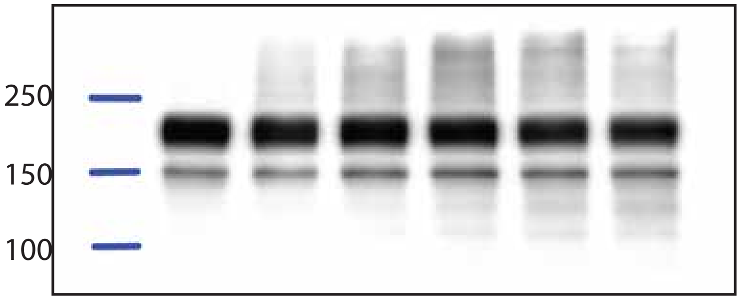

Figure 2a, M28-D5, PDGFRβ

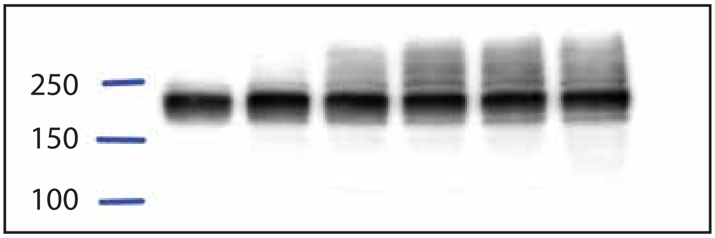

Figure 2b, 2054E, PDGFRβ

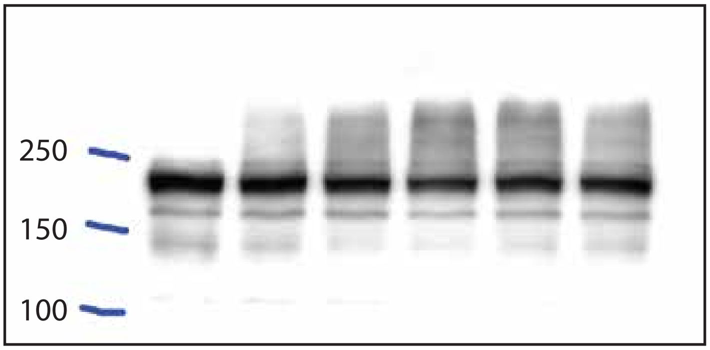

Figure 2a, M28-D5, α-tubulin

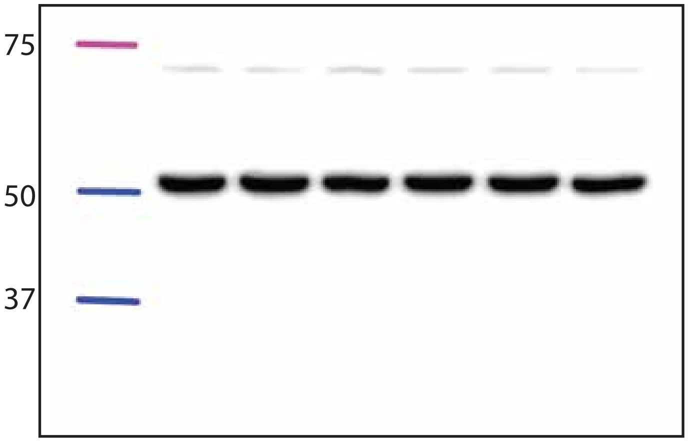

Figure 2b, 2054E, α-tubulin

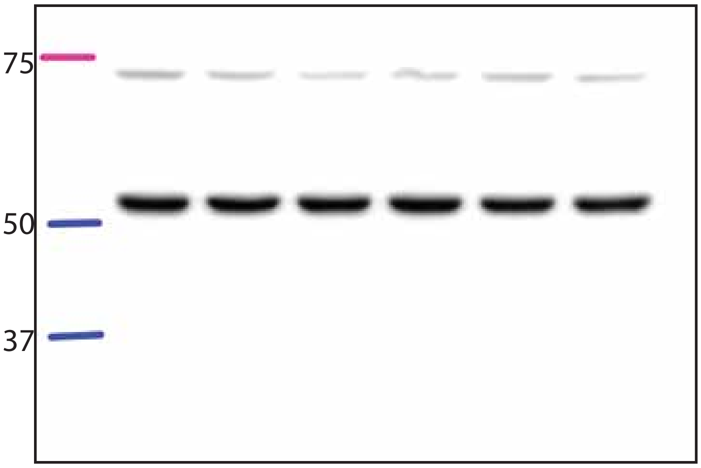

See Figure 2 in the main text for lane labels.

Supplementary Figure S1, Full-length images of cropped immunoblots in figures, continued

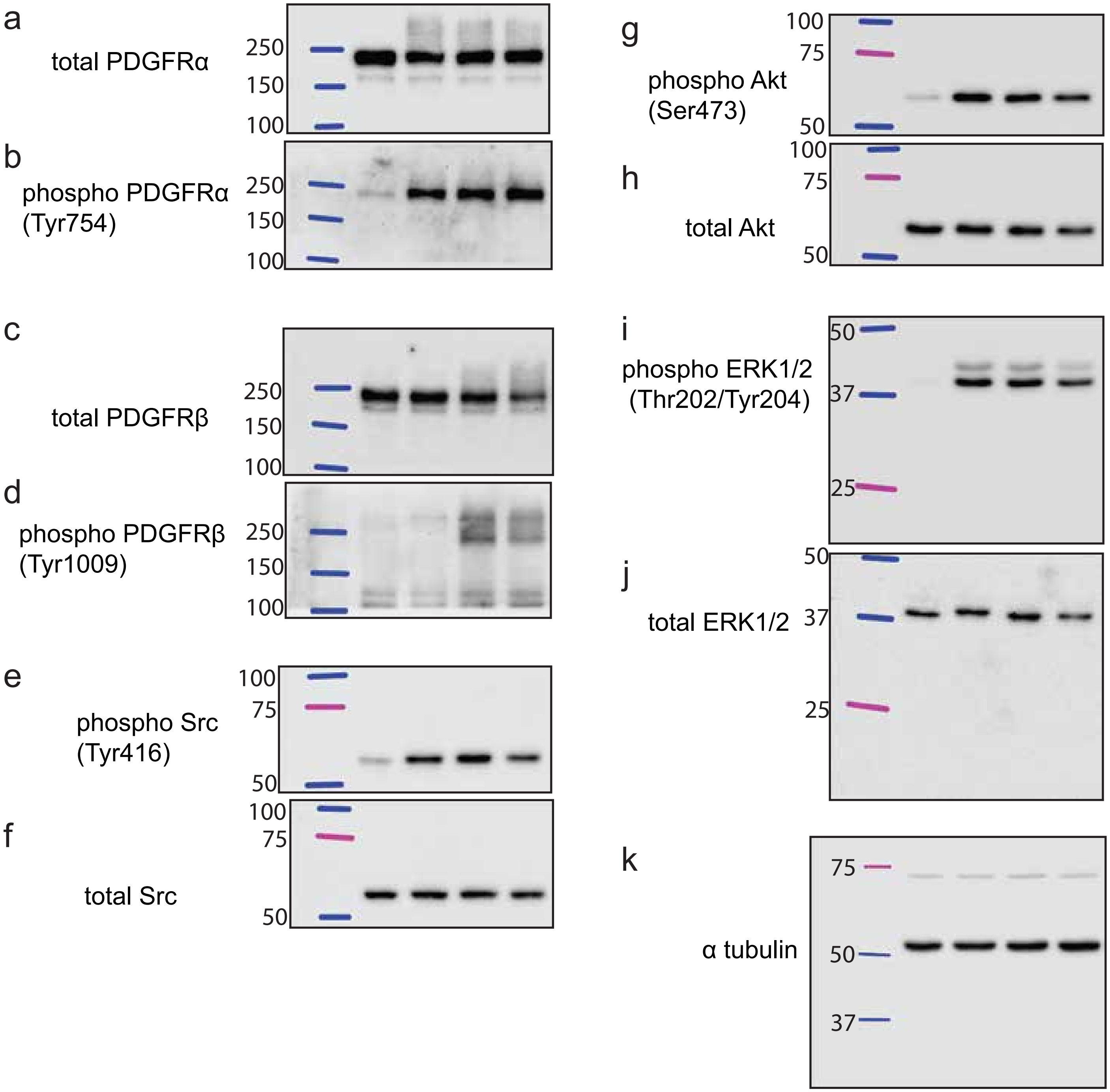

See Figure 3 in the main text for lane labels.

Supplementary Figure S1, Full-length images of cropped immunoblots in figures, continued

Figure 5b, M28-D5, PDGFRα

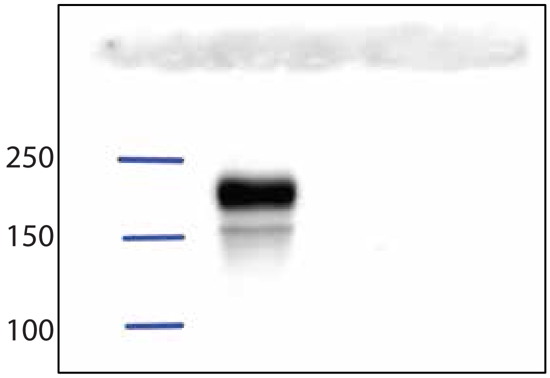

Figure 5b, 2054E, PDGFRα

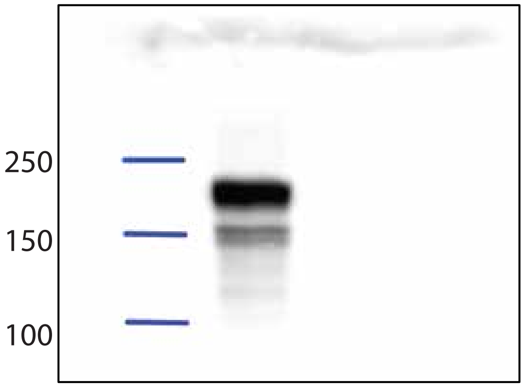

Figure 5b, M28-D5, PDGFRβ

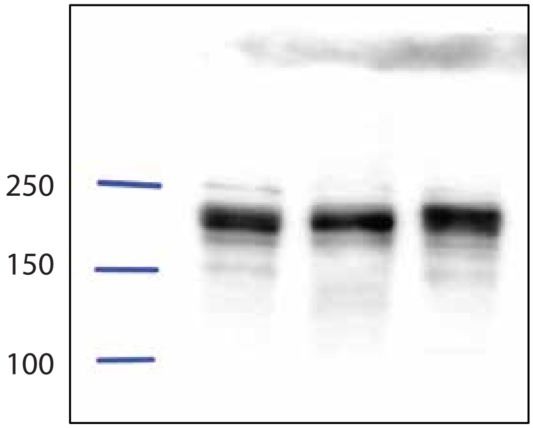

Figure 5b, 2054E, PDGFRβ

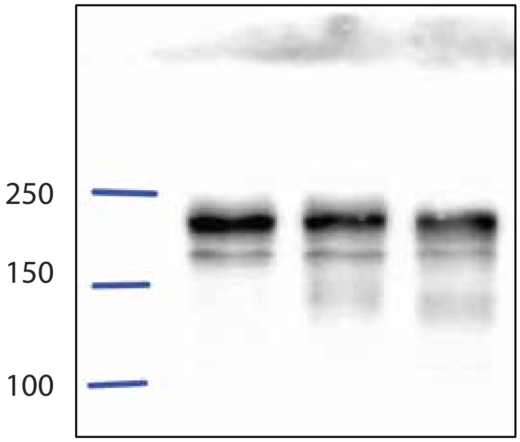

Figure 5b, M28-D5, α-tubulin

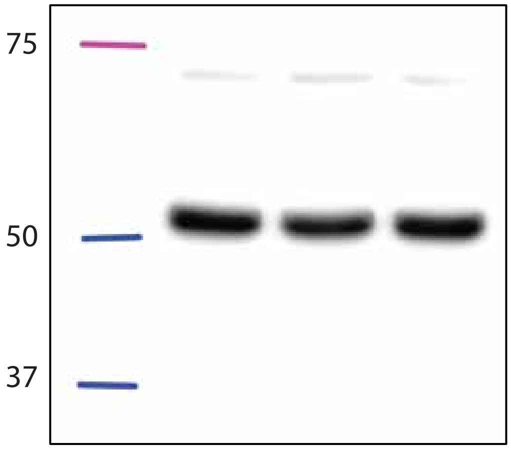

Figure 5b, 2054E, α-tubulin

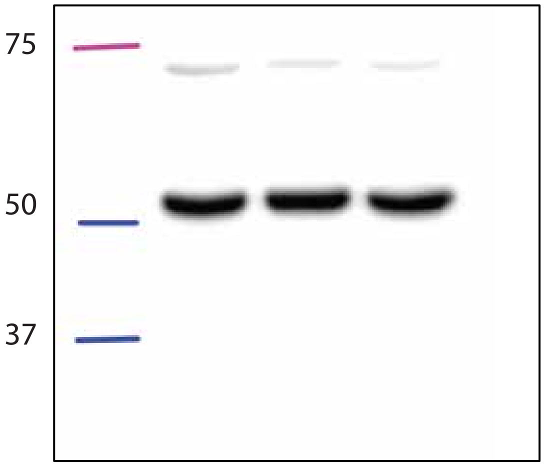

See Figure 5 in the main text for lane labels.

Supplementary Figure S1, Full-length images of cropped immunoblots in figures, continued

Figure 6a, M28-D5, PDGFRα

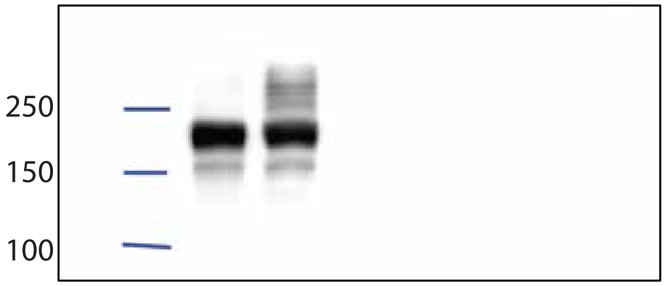

Figure 6b, 2054E, PDGFRα

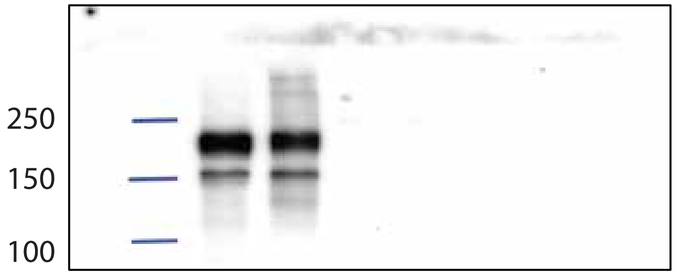

Figure 6a, M28-D5, PDGFRβ

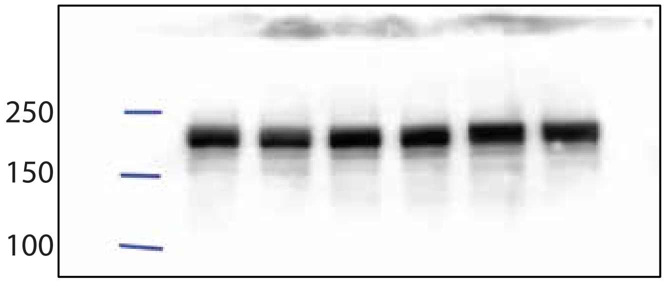

Figure 6b, 2054E, PDGFRβ

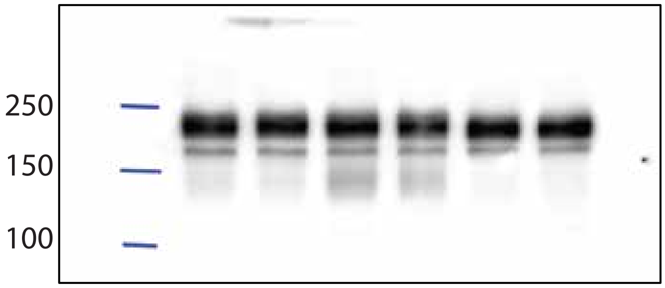

Figure 6a, M28-D5, α-tubulin

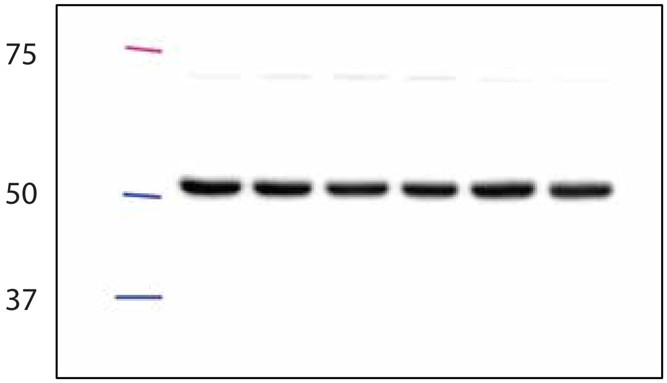

Figure 6b, 2054E, α-tubulin

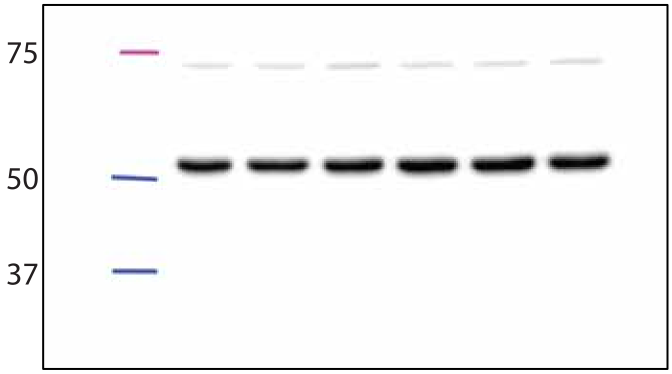

See Figure 6 in the main text for lane labels.

Supplementary Figure S1, Full-length images of cropped immunoblots in figures, continued

Figure 7a, M28-D5, PDGFRβ

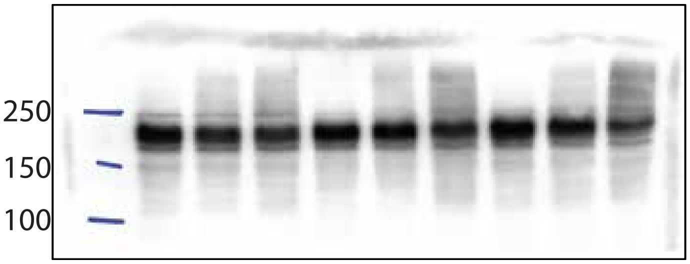

Figure 7b, 2054E, PDGFRβ

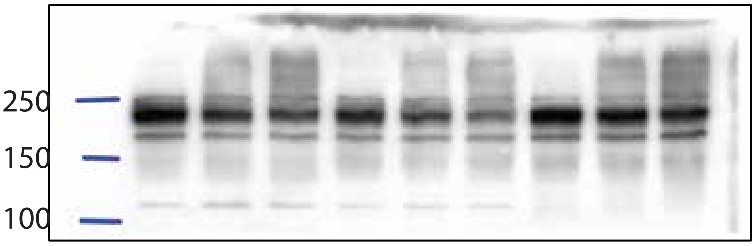

Figure 7a, M28-D5, α-tubulin

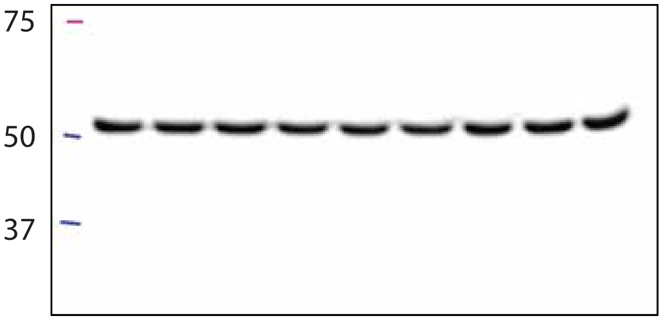

Figure 7b, 2054E, α-tubulin

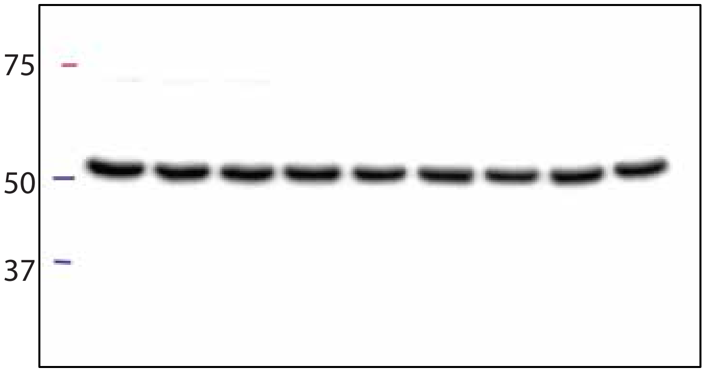

See Figure 7 in the main text for lane labels.

Genetic analyses in mouse fibroblast and melanoma cells demonstrate novel roles for PDGF-AB ligand and PDGF Receptor alpha

Julie L. Kadrmas[1,2]*, Mary C. Beckerle[1,3]* and Masaaki Yoshigi[4]*
[1]Huntsman Cancer Institute, [2]Department of Oncological Sciences, [3]School of Biological Sciences, [4]Department of Pediatrics at the University of Utah, Salt Lake City, UT 84112

* corresponding authors: julie.kadrmas@hci.utah.edu, masaaki.yoshigi@hsc.utah.edu, mary.beckerle@hci.utah.edu

SUPPLEMENTARY INFORMATION

Figures S1
